# New York Academy of Medicine—Section in Orthopaedic Surgery

**Published:** 1899-07

**Authors:** 


### New York Academy of Medicine—Section in Orthopaedic Surgery.

Meeting of March 17, 1899.

### LATERAL DEVIATION OF THE SPINE AND PES CAVUS IN FREDREICH'S ATAXIA.

Dr. W. R. Townsend presented a boy, 20 years of age. Since an attack of scarlatina at the age of 7 his nutrition had been very poor. The first signs of ataxia were an unsteady gait and inability to keep from falling if pushed. For the past seven years he had had frequent pain in the knees. Lateral curvature of the spine appeared three years ago, and has steadily increased, a long curve to the right extending from the ninth dorsal vertebra downward, with rotation. A plaster of Paris corset had been applied with moderate suspension. There was pes cavus, but no equinus. The gait was markedly ataxic. Standing with feet separated and eyes closed there was swaying of the body. The patellar reflexes were lost. Speech was slow. There was nustagmus, but no Argyle-Robertson pupil.

Dr. J. Collins said that it was a clinically typical case. In addition to disease of the posterior columns there was sclerosis of the lateral parts of the cord, including the direct cerebellar tracts shown in persistent efforts of the patient to balance himself and producing the peculiar condition found in every case and heretofore undescribed, aptly named the fork-prong condition of the extensor tendons, the feet being in continual balancing action with the tip of the toes digging into the substance of the floor. The dynamic deformities, which later became static, were the result of some connate lack of development in the anisotropous muscular substance. The deformity might be explained by postulating the existence of some congenital incapacity of development, some abnormal condition of the proton of the muscular substance. The disease was progressive, and usually uniformly so, and might extend through half a normal life-time. There was something attractive about the theory that some fibres of the spinal cord might have suffered death fifty or sixty years before the normal time, a death without active inflammatory or degenerative changes and akin to that which attended senility. The plaster of Paris corset could have no influence on the disease, but it had, in his experience, contributed to comfort. A potent agent in restoring the function of the muscles was the re-education of the extremities. The patient might be so taught that in a few months he would be able to walk into the room without perceptible disturbance of gait.

Dr. S. Ketch said that the association of nervous disease with lateral curvature was suggestive. Many features of the latter affection could not be explained except by the presence of some prior defect in the nervous system. The case came near being an argument for the neural etiology of lateral curvature.

Dr. H. L. Taylor said that the argument was not convincing. The coincidence of nervous diseases could not establish the neuropathic origin of lateral curvature, which we saw also in collapse of the lung without rating pulmonary diseases as an important etiological factor.

Dr. A. B. Judson said that a nervous origin was not altogether improbable from the observation that the curvature appeared to be due to inability of the muscles to sustain weight while the muscular failure seemed to be the result of faulty innervation.

Dr. Ketch said that in the absence of a demonstrable etiology he would adhere to the opinion that a large number of cases were caused by an antecedent fault in the nervous system.

### CONGENITAL DEFORMITY OF THE LOWER EXTREMITY.

Dr. Ketch presented a girl baby two months old, with great bony deformity of the right lower extremity. There was shortening and twisting of the upper end of the femur, and all the bones were smaller than those of the left leg. The fibula was indistinct, giving only the feeling of cartilaginous hardness. The place of the patella was marked by a slight immovable eminence. There was marked equinus with inversion, the motion of the knee was greatly limited in extension and the spine was slightly deviated to the left in the lower dorsal region. There was dimpling and adhesions of the skin to the outer side of the lower end of the femur. The head had presented in an easy labor with the cord wound around the body so that it held the right foot on the left buttock, "so tightly bound there was no blood in the leg until an hour." The cause of the deformity was evidently retention of the parts in the fœtal position by pressure of the cord, the limb being unable to escape and develop normally.

Dr. Taylor said that the bones were all present, but the fibula seemed to be fully developed only at its lower end, and the deformity of the foot was not the one usually associated with absent fibula. In these cases some bone was usually lacking or rudimentary.

Dr. V. P. Gibney said that the clear history sufficiently explained the cause of the deformity. He recalled the case of a child born with dislocation of both hips and both knees, arrest of development being found at the knees, and double club-feet of an exaggerated type. The elbows were defective and the movements of the shoulders rather limited. Repeated operations had been required, with plaster of Paris retention, and as a result the patient had for several years been walking about and going to school without apparatus or any other assistance. He had under observation another child with prenatal amputation of several fingers and double club-foot with arrested tibial development. The fibulae being very much elongated he had divided them obliquely about two inches above the malleoli, and slipped the distal portion up on the proximal, thus bringing the foot into very good position.

CONGENITAL LATERAL CURVITURE OF THE SPINE.

Dr. R. Whitman presented a girl 7 years of age, whom he had first seen when she was 9 months old. She then presented a well marked rotary lateral curvature of the spine that had been noticed by her mother immediately after birth. In spite of the application of braces and manipulation the curvature grew worse rapidly, until two years ago, when the tilting of the pelvis was so extreme that there appeared to be marked inequality in the length of the legs. The degree of the deformity was seen in a Roentgen picture. Since that time she had been under treatment by irremovable plaster jackets, applied with as much corrective force as could be borne, with most gratifying results. The pelvis became level and the limp had disappeared. The spine had become flexible and its deformity had been in great part corrected. This method of forcible correction and retention in severe curvatures of this class in young children appeared to offer the best chance of ultimate success.

Dr. G. R. Elliott said that the child's head, shoulders, hips and lower extremeties were developed far beyond the thorax as one of the results of two years' encasement. The plaster of Paris jacket is advisable in proper cases, but it should be renewed once in three months, and should be removed at least weekly to permit breathing exercise and massage.

Dr. R. H. Sayre said that bad effects do not necessarily follow prolonged treatment in the plaster jacket. He recalled the case of a boy affected with rachitic lateral curvature who was unable voluntarily to stand in an upright position. He was kept in solid plaster of Paris for a period of three years. When the jacket was removed treatment to develop the muscles restored them to as good condition as the muscles of the rest of the body.

Dr .Taylor said that he did not hesitate to immobilize joints and their acting muscles for years if necessary to arrest disease. He had never seen a case in which, after such treatment, the muscles were not developed to the limit imposed by joint motion. It had been demonstrated clinically that when motion was restored to knees anchylosed for many years the muscles assumed their functional activity.

Dr. Ketch said that atrophy of muscles and stiffness of joints caused by the application of plaster of Paris or a brace were of no serious moment, and were followed by no ultimate bad effect.

Dr. Elliott believed that permanent injury followed prolonged confinement of children in plaster of Paris forcibly applied. He had a patient under treatment who had been thus treated for seven years, and was, as a result, a hopelessly bed-ridden invalid. It might be an exceptional case, but with a neurasthenic temperament and enfeebled muscles present the injury would extend beyond the possibility of rehabilitation. The muscles might revive, but the bones and cartilage of the thorax would be atrophied to the ultimate impairment of the heart and lungs.

Dr. Townsend suggested that the same improvement might have been secured if the jacket had been replaced by a firmly applied corset, whose occasional removal would have permitted the employment of massage.

Dr. Whitman said that the child had worn a brace, which the mother was instructed to remove and give the child massage, but until the jacket was applied as described the patient grew steadily worse.

### THE EFFECTS OF GYMNASTIC EXERCISES IN REMEDYING THE DISPLACEMENT OF THE HEART IN LATERAL CURVATURE.

Dr. T. E. Satterthwaite presented a paper to the effect that the malposition of the thoracic and abdominal viscera which attended well advanced cases of lateral curvature might be considered as a constant menace to health, and it could be inferred that the thoracic pain of this affection, due in some patients to neurotic conditions, was due in others to the faulty position of the heart, which was generally displaced towards the concavity. He presented a patient, a young women 24 years of age, affected with lateral curvature toward the right in the dorsal region of the spine. The pelvis was tilted and the left breast was prominent. When first seen in the summer of 1898, she was pale, anaemic and short-winded. The heart's action was weak, and the apex one inch to the left of the nipple. After three month's treatment with resistant exercise, electricity, gymnastics and massage the anaemia was corrected, the heart's action was improved and the apex was well to the inner side of the nipple line. Its change in position was traced in diagrams taken successively during the progress of treatment. Two other patients were presented with similar histories and with diagrams showing the migration of the apex during treatment and coincidently with the improvement in the general and local condition of the patient. These patients illustrated in person a long series of appropriate exercises, in many of which indicated muscles were called into action by resistance applied by a medical attendant. The exercises were taken by the patient standing erect, leaning against a support, sitting, recumbent, semi-recumbent or suspended by the hands. In the majority of cases there was an advantage in combining force for the reduction of the deformity with some of the prescribed exercises and manual force should be applied without the assistance of mechanical apparatus. Double pressure should be made when practicable, one hand being placed upon the dorsal convexity and the other on the lumbar convexity, each pressing towards the spine. As a rule tonics or nutrients were required; iron, strychnine, cod liver oil and malt extracts. Massage of the muscles of the back was a valuable adjuvant, and the Faradic current might be applied successfully during the entire course of the treatment, employed so as to contract actively the muscles of the back. An effort should be made, where practicable, to do away with the spinal brace, which should be advocated only as a temporary expedient or in cases in which all other measures had failed. By pursuing a more thorough and painstaking course than that commonly in vogue the heart, and with it the lungs, and in time the abdominal viscera, might in a measure be restored to their natural position.

Dr. Sayre said that inspection of a preparation of lateral curvature showed that suffering from impeded action of the heart and lungs probably attended cases of well marked deformity. As a rule, however, such patients were not prone to die of disease of the heart or lungs, and, although perhaps somewhat disturbed, they lived to a good old age. He had seen distinct relief of shortness of breath from treatment by exercises, and patients in whom the rapidity of the heart beat had been materially reduced. In one case the pulse rate came down from 120 to 90 when suspended, and 106 when in a plaster of Paris jacket. He had a patient under observation in whom a murmur distinctly audible at some distance and in certain positions of the body, and sounding very much like a tin whistle, had disappeared under the influence of exercises.

Dr. Satterthwaite said that the murmur has been probably due to anaemia and a flabby condition of the chambers and ostia of the heart. He did not think that cardiac displacement in these cases gave rise to abnormal sounds, extrinsic or intrinsic.

Dr. H. S. Stokes said that he thought it was very difficult to say whether the position of the heart had changed or not. It was the opinion of some observers that the heart could not be accurately mapped out during the life of a normal chest. In a chest deformed by lateral curvature the element of possible error must certainly be a large one. In his observation the result of treatment had been an improvement in the general condition of the child and the prevention of an increase of the deformity rather than an obliteration of the curvature.

Dr. Satterthwaite said that while many physicians among the Germans and English rejected methods of mapping out the heart, in this country observing the heart in this manner was accepted as practicable and important. He believed that it was easy to determine the position of the apex by the impulse and also by the use of the stethoscope.

Dr. J. Teschner said that the heart could not be directly affected to an appreciable extent unless the deformity was so great as to crowd and displace it. He had not mapped out the heart in his cases but its change or position as the result of treatment by heavy gymnastics had been obvious. In a girl 19 years of age a very severe rotary lateral curvature of at least 10 years' duration was combined with cardiac trouble dating from acute articular rheumatism and peri- and endo-carditis at the age of 4. There was marked hypertrophy and dilatation, a double aortic murmur, a double mitral murmur and a very decided murmur over the pulmonary with the second sound. The murmurs were very widely transmitted. Dyspnœa was marked. Slight cyanosis at rest became marked on the slightest exertion. The heart had been growing rapidly weaker, oedema had appeared and her physician believed that she would live only a year or two longer. Beginning with very gentle exercises, in six months she was practicing heavy gymnastics and her physician expressed surprise at her improved condition. He found the heart smaller and changed in its relative position to the chest wall and none of the murmurs except the pre-systolic mitral transmitted to the side and back as before. Dr. Teschner believed that the deformity could be reduced by the voluntary and resisted efforts of the patient and not by external force. Electricity and massage were valueless when compared with voluntary exercise. The more the patient exercised the muscles through the medium of the will the greater would be the benefit. He thought that the exercises described and exhibited fell far short of what was required and that their effect in severe cases would be like that of an infinitesimal dose of a drug whose full physiological effect was desired. He thought that one curve could not be modified without a corresponding effect on the compensating curve. The trouble was not the deflection of a single vertebra but of several, leading to the production of the sigmoid deformity.

Dr. Satterthwaite agreed that the different curves should be considered together as making up the deformity and added that in the treatment the muscles should be also considered together, as it was impossible to exercise or develop one muscle or group without acting on all the muscles of the region.

Dr. Taylor said that while electricity and massage were good they were not sufficiently good to cure lateral curvature. Reliance should be chiefly on muscular training and suitable apparatus. He would welcome any possible way of dispensing with apparatus which, useful in selected cases, left much to be desired. The hygiene of the patient was of great importance. The physician should regulate the food, schooling, exercise and rest. Piano playing was a pernicious occupation for a patient with a weak back. It should be moderated and usually stopped. One of the things which had held us back in the treatment of this affection was the difficulty in measuring and recording changes which take place. The position of the heart might perhaps in some cases be a useful indication. Measurement of the height from time to time was more easy and likely to furnish more reliable observations.

Dr. Satterthwaite said that he was in the habit of recording the height as a routine matter, but in growing children such measurements might be misleading.

Dr. Ketch said that apparatus was of value in retaining the improvement gained through exercise, which when properly conducted produced a good effect on the deformity and indirectly on the condition of the heart, for there was no doubt that the changes in the vertebrae themselves and in the chest walls and the diameter of the thorax gave rise to changes in the viscera. As long as rotation persisted no case of lateral curvature could be said to be really cured. This was always a menace and liable to increase and was the most difficult element to control. The bony changes which followed the muscular changes also made the treatment of lateral curvature very difficult. Curvature depending on simple muscular weakness was the easiest to control, but these were not cases of true rotary lateral disease. Each man should work out his own ideas in regard to the question of exercises, remembering that no form of treatment would be of the slightest value unless it was continued for a long time.

Dr. Satterthwaite agreed that not all cases were suitable for the treatment which he had described. It could not easily be made successful in the case of out patients, especially those who lived far away and thus were unavoidably irregular in their attendance. The patients presented were all improving in general condition, the spine was gradually moving forward towards the normal position while the heart in each had taken an improved position.

### THE PELVIC REST.

Dr. Townsend exhibited a simple apparatus to facilitate the application of a plaster of Paris spica to the hip. It held the pelvis and thigh up so that the roller might be conveniently passed between the patient and the table, and when the application was made and set the thin steel shelf on which the pelvis rested might be readily withdrawn from between the bandage and the patient. It was similar in action to the rest shown by Dr. T. H. Myers at the last meeting of the section. The standard or vertical part, $6x1\frac{1}{4}x\frac{1}{4}$ in. was forged at its upper end into a thin horizontal shelf 10x2 in. and at its lower end it was bent at right angle to form the bar, 11 in. long, which rested on the table. Two cross pieces, 11 in. long, of light steel were provided with mortises by which they could be removed for packing or adjusted by sliding them along on the bar until they were in position to hold the apparatus firmly, without rocking, on the table.

